# National Rugby League athletes and tendon tap reflex assessment: a matched cohort clinical study

**DOI:** 10.1186/s12891-016-1305-3

**Published:** 2016-11-04

**Authors:** James Maurini, Paul Ohmsen, Greg Condon, Rodney Pope, Wayne Hing

**Affiliations:** 1 Physiotherapy, Bond University, 2 Promethean Way, Robina, Queensland Australia 4226; 2 Gold Coast Titans, Gold Coast, Australia

**Keywords:** Tendon tap reflex, Stretch reflex, Elite athlete, Monosynaptic reflex

## Abstract

**Background:**

Limited research suggests elite athletes may differ from non-athletes in clinical tendon tap reflex responses.

**Methods:**

In this matched cohort study, 25 elite rugby league athletes were compared with 29 non-athletes to examine differences in tendon reflex responses. Relationships between reflex responses and lengths of players’ careers were also examined. Biceps, triceps, patellar and Achilles tendon reflexes were clinically assessed.

**Results:**

Right and left reflexes were well correlated for each tendon (r_S_ = 0.7–0.9). The elite rugby league athletes exhibited significantly weaker reflex responses than non-athletes in all four tendons (*p* < 0.005). Biceps reflexes demonstrated the largest difference and Achilles reflexes the smallest difference. Moderate negative correlations (r_S_ = −0.3–0.6) were observed between reflex responses and lengths of players’ careers.

**Conclusions:**

Future research is required to further elucidate mechanisms resulting in the observed differences in tendon reflexes and to ensure clinical tendon tap examinations and findings can be interpreted appropriately in this athletic population.

## Background

Assessment of tendon reflexes is an important part of a clinical examination in athletes. There is a close relationship between muscular strength and neurological function, which requires examination of tendon reflexes in order to develop an accurate picture of the athlete’s function. Clinically, reflex responses to tendon tap are subjectively graded from 0 (absent reflex) to 4 (clonus response) with a grade of 2 being considered clinically normal [1] and the tendon tap reflex test has demonstrated reasonable test-retest reliability [2]. However, it is currently not clear whether reflex responses differ between athletes and non-athletes.

A systematic review of the published literature on tendon reflexes in athletes completed by the authors revealed insufficient good quality research, so that no firm conclusions could be drawn regarding the relative strength of reflex responses to tendon tap in an elite athletic population when compared to a non-elite population. The methods used in the small number of identified studies [2–6] were rated as being of poor quality when critically appraised using the Downs and Black checklist [7].

Of particular interest, it was noted from the available literature that certain factors, which are common in the elite sporting population, can have an impact on tendon reflex responses. Research on amateur power trained athletes [5, 8, 9] and endurance trained athletes [3, 5, 9, 10] found exercise intensity, the type of contraction, training history, recent stretching and fatigue were all such factors. National Rugby League (NRL) players are primarily power-trained athletes but also complete endurance and a variety of strength training techniques. Further clinical observations during player assessment and management have suspected the aforementioned. The unique demands of the NRL provide increased exposure to these reflex-modifying factors. It is therefore our hypothesis that professional rugby league athletes have a possible diminished reflex response to tendon tap examinations. Knowledge of any normal differences between elite athletes and the general population in reflex responses to tendon tap is important for clinicians working with elite athletes, as it will affect their interpretation (as normal or abnormal) of reflex responses observed with a tendon tap during clinical assessment of elite athletes. This clinical interpretation of tendon reflex responses may also be important, diagnostically.

The current study was therefore developed to address the evident gap in the literature surrounding reflex responses to clinical tendon tap examinations within an elite athletic population - specifically National Rugby League (NRL) players. The primary research aim was to examine whether NRL athletes had altered reflex responses to tendon tap when compared to an age- and gender-matched non-athlete cohort. A closely-linked secondary aim was to ascertain if the length of a player’s career was associated with their clinically-assessed strengths of reflex responses to tendon tap.

## Methods

### Setting and research design

The research was conducted in southeast Queensland, Australia, within a professional Rugby League organization and Bond University. The research used a matched cohort study design involving a cohort of professional elite Rugby League players and a similar-sized cohort of participants of the same sex and with equivalent mean age and age range, who were not elite athletes. The study was approved by the Bond University Human Research Ethics Committee (protocol number RO1905).

### Participants

All athlete participants were recruited via information sessions conducted within a single professional rugby league organization prior to the beginning of the 2015 season. Non-athlete participants were recruited via flyers posted at Bond University in Robina, Queensland, Australia. In each case, informed consent was obtained before participation in the study.

All participants were required to meet the following inclusion criteria: 1) healthy male individual between the ages of 18 and 35; 2) no current muscle or tendon injuries in the areas to be examined. In addition, the age criterion for non-athlete participants was further limited to ensure the final cohort of non-athletes was closely age-matched to the cohort of elite athletes, and potential non-athlete participants were excluded if they had a history of elite athletic training. The latter exclusion criterion resulted in only one person being excluded from participation in the non-athlete cohort, due to a history of playing semi-professional sport.

The final non-athlete cohort consisted of 29 male university students, and the elite athlete cohort consisted of 25 professional Rugby League players (Table 1).

**Table 1:** Participant characteristics (mean +/- SD)

	Non-athlete (*n* = 29)	Athlete (*n* = 25)
Age	24.14 years ± 2.95 years	24.36 years ± 2.97 years
Height	180.07 cm ± 5.26 cm	187.08 cm ± 20.27 cm
Weight	80.83 kg ± 12.17 kg	94.66 kg ± 10.75 kg
Years Pro	0 ± 0 years	3.8 ± 3.23 years
NRL games played	0 ± 0 games	55.68 ± 62.46 games

### Data collection

Participants were assigned a number for the duration of the study to maintain anonymity. Both the athlete and non-athlete cohorts underwent the same testing procedure and were assessed by the lead researcher within a single session for each group. Examination of the athlete cohort was completed at the rugby club’s facilities, to mimic a realistic training environment. Data collection from these athletes took place at 7 am following two full days of scheduled rest and prior to any training sessions that day. The non-athletes were examined during a 9 am session at Bond University.

A standard, clinical tendon hammer was used in order to elicit a reflex response in the participants. Each tendon was located and tapped directly, 2–4 times with the hammer, approximately 2–3 cm from its distal insertion into the bone. An exception to this was the Biceps tendon, which was indirectly tapped by striking the researchers thumb, which was placed over the tendon. The tendons were not tapped again until the limb had returned back to its resting position and any movement/muscular activity had ceased.

Participants were first seated on a plinth with the legs hanging over the edge and were asked to relax. The testing positions used to examine the biceps, triceps and patellar reflexes while in this seated position were in accordance with clinical recommended assessments [11]. The achilles tendon was assessed with the participant in prone, with the knee bent to 90 degrees of flexion. This position was believed to be more sensitive than the standard seated position, based on clinical experience of the research team.

The response of each tendon was graded from 0 to 4, which is the current clinical standard for scoring tendon tap reflexes [1]. Responses were subdivided into the following categories based on the median reflexes of the participant. Clinical scores of 1–1.5, 2–2.5 and 3–4 were considered hypoactive, normal and hyperactive respectively. All data collection was completed in a single session to avoid heterogeneity in reflex responses that might be associated with variability in preceding training loads. There was no follow up required in this study, and therefore no individuals were lost to follow up.

### Data analysis

Spearman’s rank-order correlation coefficients were calculated to determine the extent to which left and right tendon reflex responses were correlated for each of the included tendons. Noting the ordinal nature of the data, Mann–Whitney U tests were conducted to examine differences in the distributions of tendon reflexes between the athlete and non-athlete cohorts.

Further Spearman’s rank-order correlation coefficients were calculated in the athlete cohort to examine the magnitude of the correlations between tendon reflex scores and: (1) player age; (2) the number of years for which professional sport had been played; and (3) the number of professional-level NRL games that had been played. All data analysis was performed using SPSS version 22 [12].

## Results

Calculated Spearman’s rank-order correlation coefficients indicated a high correlation between left and right reflex scores for biceps (rho = 0.689, *p* = <0.01), triceps (rho = 0.718, *p* = <0.01), patella (rho = 0.851, *p* = <0.01) and Achilles (rho = 0.760, *p* = <0.01) reflexes. On this basis, the Mann–Whitney U tests were completed using the median tendon reflex scores derived from the left and right tendons, for each included tendon. The Mann–Whitney U tests indicated significant differences between the athlete and non-athlete cohorts in the distributions of tendon reflex scores, for all of the tendons assessed (Table 2).

**Table 2:** Results of Mann–Whitney *U* test comparing distributions of median right/left tendon reflex scores between athlete and non-athlete cohorts

Key finding	Significance level
Distributions of *biceps tendon reflex scores* differed significantly between cohorts	*p* < 0.001
Distributions of *triceps tendon reflex scores* differed significantly between cohorts	*p* < 0.001
Distributions of *patella tendon reflex scores* differed significantly between cohorts	*p* = 0.003
Distributions of *Achilles tendon reflex scores* differed significantly between cohorts	*p* = 0.001

**Fig. 1:**
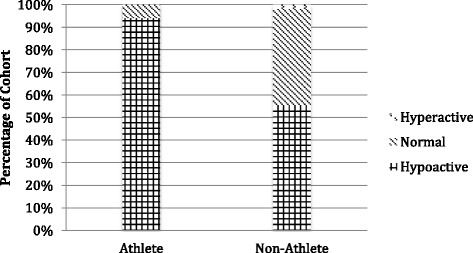
Biceps tendon median reflex response

**Fig. 2:**
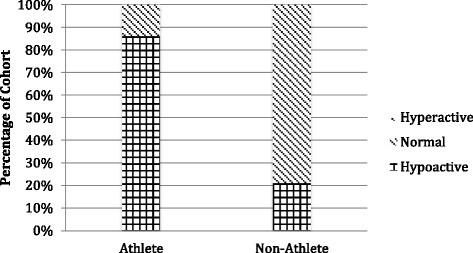
Triceps tendon median reflex response

**Fig. 3:**
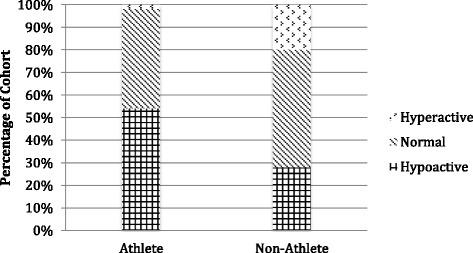
Patellar tendon median reflex response

**Fig. 4:**
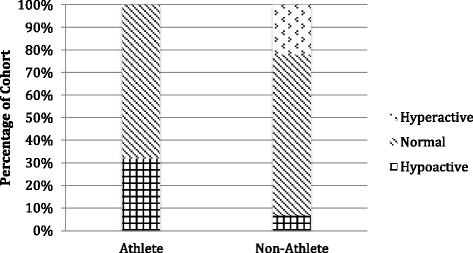
Achilles tendon median reflex response

The reflex scores for the non-athlete and athlete cohorts are shown graphically in Figs. 1, 2, 3 and 4. These comparative frequency distributions for the two cohorts, presented separately for each tendon, demonstrate clear differences between the cohorts, in all tendons, in the distributions of tendon reflex scores. In each case, the athlete cohort tended to have a higher proportion than the non-athlete cohort of hypoactive tendon reflex scores (median reflex scores of 1.5 or less). It is important to note that no participant in this study received a score of 4 (clonus) for any of the tendons that were assessed.

The biceps tendon reflex distributions (Fig. 1) were such that in 43 % of the non-athlete participants, the biceps tendon reflex was assessed as normal (median reflex score of 2 or 2.5), compared to just 6 % of athlete participants. The athlete response distribution demonstrates a shift in median reflex responses towards a diminished or absent reflex (median reflex score of 1.5 or less). Sixteen percent of the non-athlete cohort exhibited absent biceps tendon reflexes in at least one upper limb. This percentage was much higher in the athlete group, with 72 % of the cohort experiencing absent biceps reflexes in at least one upper limb.

The triceps tendon reflex distributions (Fig. 2) were similar, with 86 % of the athlete cohort having median reflex scores that reflected diminished or absent reflex responses. In the non-athlete cohort, 79 % of median triceps reflex scores were considered clinically normal, whereas only 14 % of median triceps reflex scores in the athletes were considered clinically normal. There were no participants in either cohort who received a classification of hyperactive reflexes (median reflex score of 3 or higher) for the triceps tendon.

The observed differences in reflex responses between the athlete and non-athlete cohorts were less pronounced in the lower limb. Nevertheless, diminished and absent patellar tendon reflexes (Fig. 3) were still more prominent in the athlete group, occurring in 54 % of athletes but just 28 % of non-athletes. Conversely, hyperactive patellar tendon reflexes were found in 20 % of non-athletes while only 2 % of the athlete group exhibited hyperactive patellar tendon reflexes.

Achilles reflex results (Fig. 4) show that similar percentages of each cohort exhibited clinically normal reflex responses. As with other tendons, the Achilles reflex responses featured a higher frequency of hypoactive reflexes in the athlete cohort (32 %) than in the non-athlete cohort (7 %). Conversely, hyperactive reflex responses in the Achilles tendon were much more common in the non-athletes (22 %), with none of the athletes exhibiting hyperactive responses.

The calculated Spearman’s rank-order correlation coefficients indicated that years of playing professional sport was significantly (p <0.01) and negatively correlated with all tendon reflex scores in the athletes (Table 3). The number of NRL games played was also significantly (*p* < 0.01) and negatively correlated with all tendon reflex scores except the left patella tendon reflex scores, where the correlation only reached a significance value of 0.05 (Table 3).

**Table 3:** Spearman’s Rank-Order Correlation coefficients for correlations between tendon reflex scores and both years of playing professional sport and numbers of professional games played

Reflex score	Years playing professional sport	NRL games played
Left biceps	−0.544	−0.449
Right biceps	−0.458	−0.376
Left triceps	−0.608	−0.549
Right triceps	−0.499	−0.425
Left patella	−0.281	−0.283
Right patella	−0.291	−0.250
Left achilles	−0.412	−0.393
Right achilles	−0.391	−0.371

## Discussion

The results of this study indicate clear differences between elite rugby league athletes and age- and gender-matched non-athletes in tendon tap reflex scores for biceps, triceps, patellar and Achilles tendons. In each tendon, reflex scores of the elite athletes were distributed significantly lower on the range of possible tendon reflex scores than scores of the non-athletes. In a substantial proportion of cases, the athletes demonstrated median tendon reflex scores between 0 and 1.5, indicating absent or diminished reflex responses. In many of these cases, the athletes demonstrated tendon reflex scores of zero, representing an absent reflex. These differences between the cohorts were most marked in the upper limb (biceps and triceps) tendon reflexes, with the biceps tendon reflex distributions the most different between the cohorts. Possible mechanisms for these changes will be discussed later.

Both cohorts in the present study included substantial proportions of individuals with absent or diminished reflexes, despite all participants being healthy. This finding supports the conclusion by Stam & van Crevel (1989), who stated that absent reflexes may be clinically normal in the general population. However, the current study adds to these previous conclusions the important finding that absent and diminished reflexes appear far more common in elite athletes than in age-matched non-athletes drawn from the general population. Of note, no participant from either population in the current study received a tendon reflex score of 4 for any of the assessed reflexes. This finding is in agreement with clinical guidelines [1], which indicate that a grade 4 tendon reflex response (representing muscle clonus) should be considered abnormal in populations examined in the present study.

The observed high, but not perfect, correlations between tendon reflex scores for the same tendon on the right and left sides of the body in the current study suggest that asymmetry in tendon reflex responses occurs but may not be as common in the populations sampled in the current study as previous literature [2, 6] has suggested.

Low to moderate correlations were observed in the current study between tendon tap reflex scores and the lengths of player careers, measured in terms of both the numbers of professional NRL games played and the numbers of years spent playing professional NRL. There appears to be a possible dose response relationship with one or more factors associated with playing professional rugby league and a decrease in tendon tap reflexes. It is unclear which factor or factors associated with the length of the player’s career mediated the observed correlation between length of player career and tendon reflex responses in the current study.

### Possible causes of altered tendon tap reflexes

There are many areas within the monosynaptic reflex arc where adaptation may occur with ongoing athletic activity over the length of the player’s careers, resulting in altered reflexes. A systematic search of the literature completed by the authors revealed that specific contraction types [13], exercise intensity [4], training history [3] and recent stretching [6] may be responsible for these types of changes in muscles and muscle function, and thus alter the results of a tendon tap examination.

In one study of the effects of stretching, 50 male participants from a variety of sports had Achilles tendon reflexes assessed prior to and following 3 min of passive stretching [6]. The study’s authors found a significant 5 % decrease in Achilles tendon reflex responses and this was not affected by a subsequent 10 min treadmill run. That study showed that passive stretching has the ability to affect reflex responses in the short term. To the knowledge of the authors, there has been one study exploring the long-term effects of stretching on tendon tap reflexes [14]. Guissard and Duchateau found a 36 % reduction in Achilles tendon reflexes following just 4 weeks of training. This reduction did not coincide with the decrease in passive stiffness pointing towards a possible neural mechanism for reduction in reflex responses [14]. Given that professional athletes stretch regularly in order to maintain muscle compliance, chronic effects of stretching constitute one possible explanation for the diminished tendon reflex responses observed in the athletes in the current study and further research to examine the neural vs. mechanical mechanism, is warranted.

Another possible explanation is that the resistance training completed by elite athletes might be responsible for the altered tendon reflexes observed in the current study. Sixteen weeks of general resistance training did not significantly alter patellar tendon reflexes in a group of mixed gender subjects in one previous study [8]. There is, however, contradictory evidence regarding whether particular types of muscle contractions can elicit changes in tendon tap reflexes such as those observed in the current study. Ten days of purely concentric exercise were shown to significantly decrease patellar tendon reflex responses in untrained male subjects [13]. On the other hand, eccentric exercises in the same population did not have a significant effect on tendon tap responses [13]. So, once again it is possible that specific concentric resistance training might contribute to diminishing tendon reflex responses in athletes but further research is required to elucidate this possibility. Kaufman et al. [13] also noted that tendon tap reflexes were not influenced by exercise related muscular trauma or delayed onset muscle soreness (DOMS).

The current study showed diminished tendon tap responses in athletes for all muscle groups assessed. Similar findings were reported with a mixed gender study comparing endurance athletes with a non-athlete cohort, with regard to patellar tendon reflexes. Endurance athletes were found to have significantly diminished patellar tendon reflex force (42.3 %) when compared to the non-athlete cohort [3]. It was noted that Achilles reflexes were not significantly different between the endurance and non-athlete cohorts [3]. The similarity between these findings and those of the current study suggest that some aspects of endurance training or specific sports requirements might contribute to diminishing tendon reflex responses.

Although each theory has its merits, it is likely a combination of several factors come together to create the athletes training history. Proske and Morgan demonstrated that eccentric strength training (a component of power training) causes muscle adaption by increasing the number of sarcomeres in series and therefore causes a compliance change within the tissue [15]. These compliance changes may alter the sensitivity of the spindle fibers and cause a reduction in the reflex response of power-trained athletes as demonstrated in the current study. It has been hypothesized that postural muscles, which are primarily slow twitch motor units, have a lower reflex threshold than other muscles due to a higher concentration of muscle spindle fibers [5]. The postural muscles of the calf may be resistant to small compliance changes due to the higher concentration of spindle fibers and their consistent use as postural muscles on a day-to-day basis. This hypothesis may explain why the Achilles reflex in the present study was least affected by change within the athlete group. Other elite athletes like swimmers have been shown to have the opposite response with an increase in Achilles reflex response when compared to the general population [16]. The long term microgravity that the athletes are exposed to within the pool is believed to be a contributing factor for their reflex changes [16]. The relatively plantarflexed position of the foot while swimming may also assist in creating some compliance changes within the muscle and tendon structures.

This brief excursion into relevant literature illustrates that there are multiple interlinking factors that elite athletes encounter on a daily basis which have the potential to contribute to the diminished tendon tap reflexes observed in athlete’s in the current study. Further research is required to elucidate the effects of these and other factors that might contribute.

### Clinical importance

The findings of this study indicate that traditional clinical interpretations of scores on the standard tendon reflex grading scale [1] may not be appropriate for elite athletic populations. Clinically diminished or absent reflex scores may be normal for elite athletes and given the large proportions of athletes with diminished or absent scores, they should not be viewed as a sign of neurological injury or impairment. Tendon tap reflexes are used in the medical field to monitor patients with head injuries due to the fact that that their reflex responses will be facilitated [17]. Clinically normal reflex responses in an NRL athlete may be facilitated by a central nervous system injury. Animal models have shown that concussive head injuries have the ability to increase reflex responses above baseline [18]. Research is needed in this field in order to determine if this relationship does exist in elite sport.

Tendon reflexes are a protective mechanism which limit rapid movement and stretching of joints and surrounding tissues [19]. As mentioned previously, tissue compliance changes with training have been shown to modify tendon tap reflexes. With this in mind, it is possible that there is a relationship between injury risk and altered tendon reflexes.

### Limitations

The interesting findings of the current study only begin to explore and compare the complex nature of tendon reflexes in the clinical professional sporting environment. The largest limitation of this study is the clinical aspect of the tendon tap examination. Results are exposed to subjective bias as well as bias associated with the tap itself. More precise methods are available in the research setting but unfortunately, these are not readily available within the clinical setting. Secondly the number of taps used in the current study is less than the clinical standard of 5–6 [11]. This was modified in order to limit any reflex fatigue or CNS habituation associated with the multiple tendons being assessed in one session. This may introduce some reliability bias with regards to the scores collected. The results of this study are very specific to the populations studied and cannot necessarily be generalized to females, other age groups and other forms of elite sport. Further research is required in additional populations in order to explore clinical tendon reflexes in depth.

### Future research

The results of this study set the groundwork for many possible follow up studies in the clinical setting. Further research directed at examining the mechanisms of altered tendon reflexes would strengthen the understanding of this complex response within the professional rugby environment. Further research is also required to assess the relationships between injuries or surgery and tendon reflexes. Due to the fact that rugby league is a contact sport and altered tendon reflex responses may be a sign of neurological injury, it would be of interest to examine the relationship between altered tendon reflexes and concussion incidents. Research exploring possible relationships should be completed in a highly controlled research environment in order to remove the possible bias associated with the present study.

## Conclusion

In conclusion, it is clear that elite rugby league athletes have decreased tendon reflexes when compared to age and gender matched non-athletes. A moderate correlation also exists between the length of players’ careers and the extent of diminishing of their tendon reflex responses. There are many possible mechanisms associated with the monosynaptic reflex that may be responsible for the diminishing of tendon reflexes in athletes. It is possible that there is a dynamic relationship between multiple factors, which results in the decreased reflex responses. The findings of this study suggest that the usual interpretations of the clinical grading scale for tendon reflexes [1] may not be appropriate for elite athletes due to the fact that a clinically decreased or absent reflex may be a normal reflex for that individual. Further research is required to better understand reflex response mechanisms and to ensure appropriate clinical interpretation of the results.

## Abbreviations

DOMS: Delayed onset muscle soreness
NRL: National Rugby League
